# Vertebral Osteomyelitis Due to Granulicatella Adiacens, a Nutritionally Variant Streptococci

**DOI:** 10.7759/cureus.808

**Published:** 2016-09-28

**Authors:** Jonathan York, Christian Fisahn, Jens Chapman

**Affiliations:** 1 Neurosurgery, Swedish Neuroscience Institute; 2 Orthopedic Surgery, Swedish Neuroscience Institute; 3 Department of Trauma Surgery, BG University Hospital Bergmannsheil, Bochum, Germany; 4 Orthopedics Spine Surgery, Swedish Neuroscience Institute

**Keywords:** spine, osteomyelitis, granulicatella adiacens, streptococci, bacterial infection

## Abstract

Vertebral osteomyelitis is a common pathology affecting the spine. We present the case of a 46-year-old male who was diagnosed with progressive L2 vertebral osteomyelitis due to a rare pathogen, *Granulicatella adiacens*. *Granulicatella adiacens *is part of the normal body flora and is often difficult to culture on traditional mediums. The patient required a lateral corpectomy and posterior fixation for spinal stabilization and source control.

## Introduction

Vertebral osteomyelitis (VO) is a common entity encountered in a neurosurgical practice. VO is characterized by an acute or recurrent infection of the spine and subsequent inflammatory destruction of the bone. The most common pathogen is *Staphylococcus aureus*, followed by *Escherichia coli *[1]. Progressive destruction of the anterior spinal elements can lead to instability, necessitating surgical intervention for anterior column support and spinal stabilization as well as obtaining source control. Herein, we present the rare case of a 46-year-old male who was diagnosed with an unusual organism, *Granulicatella adiacens*, a subtype of nutritionally variant streptococci (NVS).

## Case presentation

A 46-year-old male presented with progressively worsening low back pain. His pain began after a fall onto his buttocks three weeks prior to his initial evaluation. His symptoms were aggravated by movement and alleviated by lying flat. He had little alleviation of his symptoms with over-the-counter anti-inflammatories. He denied any lower extremity pain, numbness, or weakness. His medical history was notable for diabetes and smoking. He denied illicit drug use. He did endorse undergoing a dental procedure approximately one month prior to evaluation. A written informed consent is not necessary for single case reports at our institution (Swedish Institutional Review Board). 

On physical examination, he was exquisitely tender to palpation in his upper lumbar spine. He frequently shifted positions to find comfort, and he preferred to lie down. He had no lower extremity (LE) weakness, numbness, or saddle anesthesia.

**Figure 1:**
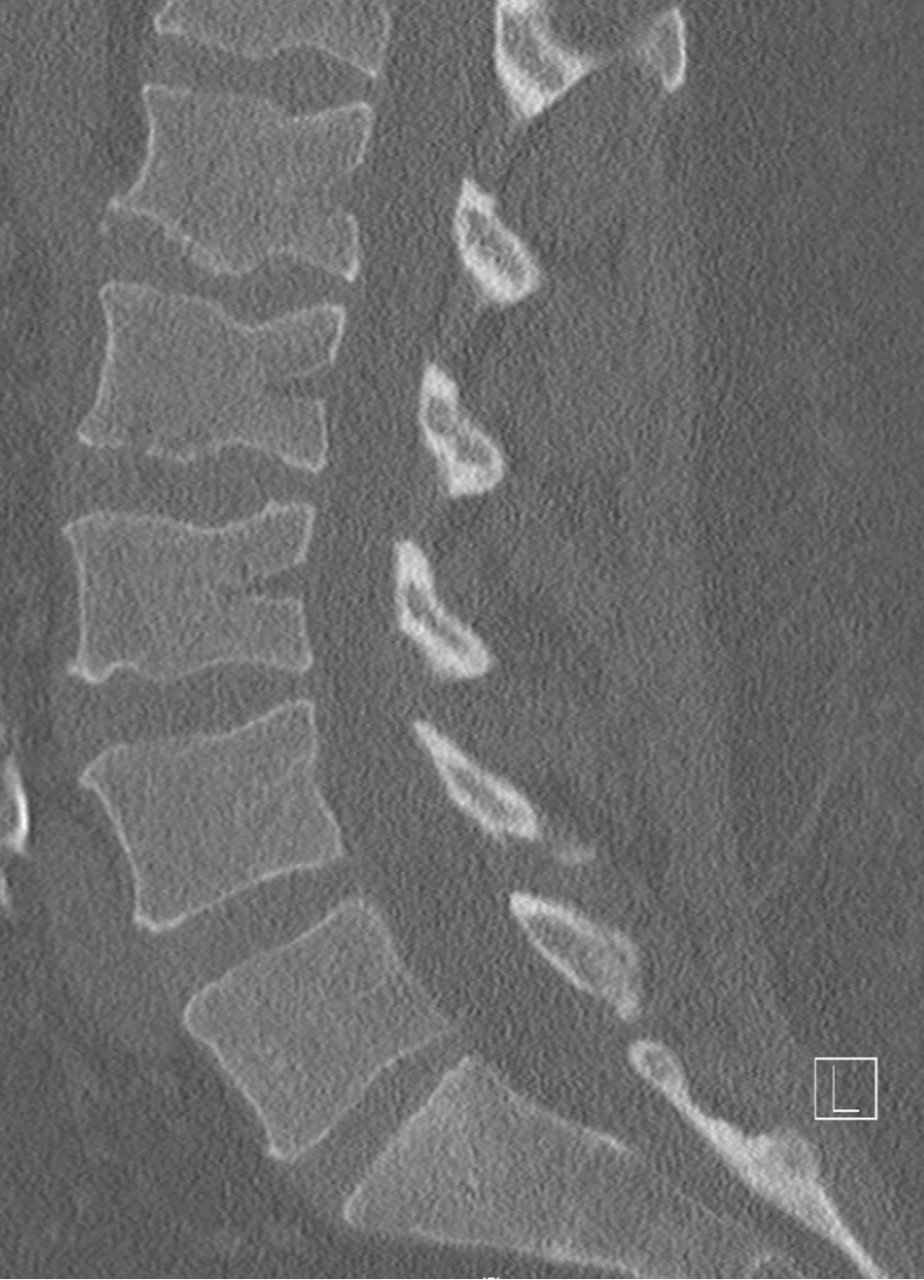
Initial CT revealing subtle irregularity of inferior endplate of L2.

**Figure 2:**
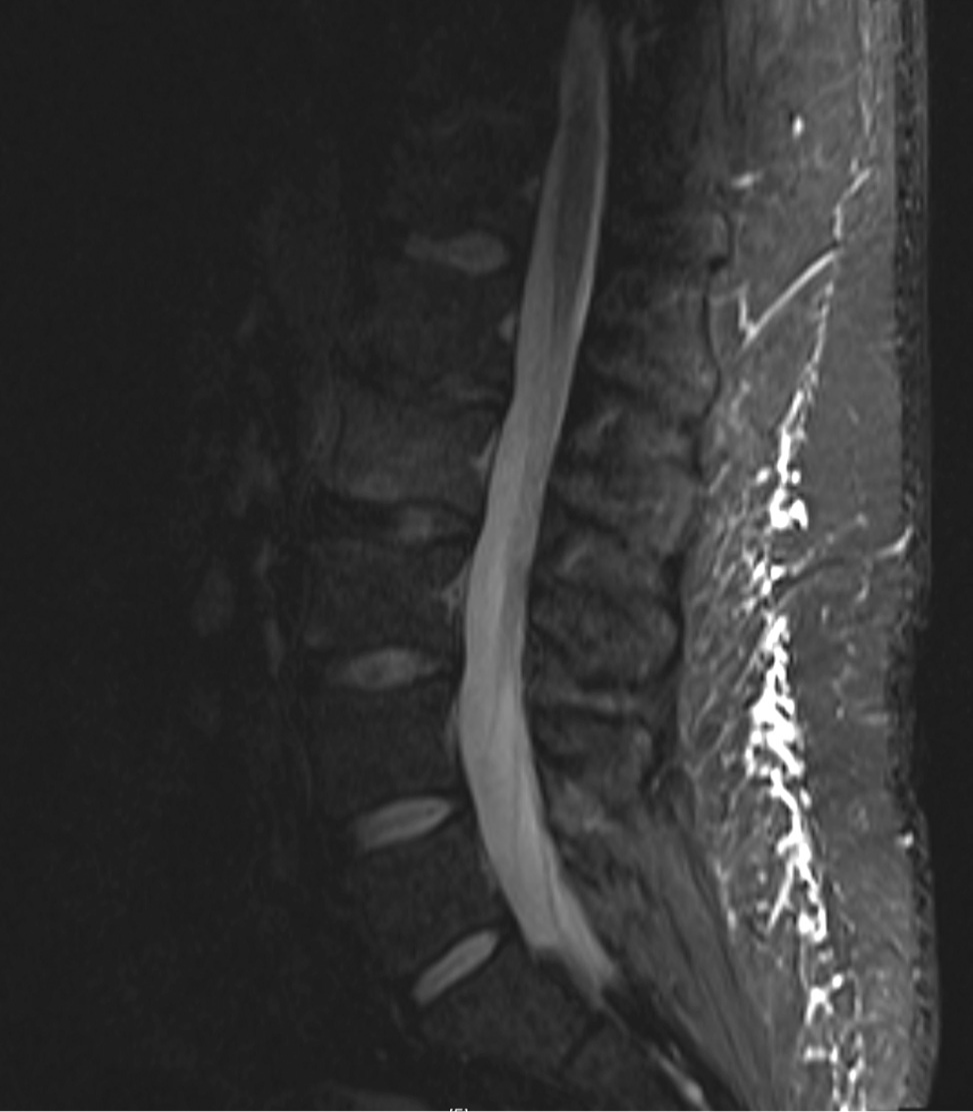
Initial MRI with contrast showing increased STIR signal change indicating diffuse bone marrow edema. STIR - short T1 inversion recovery

**Figure 3:**
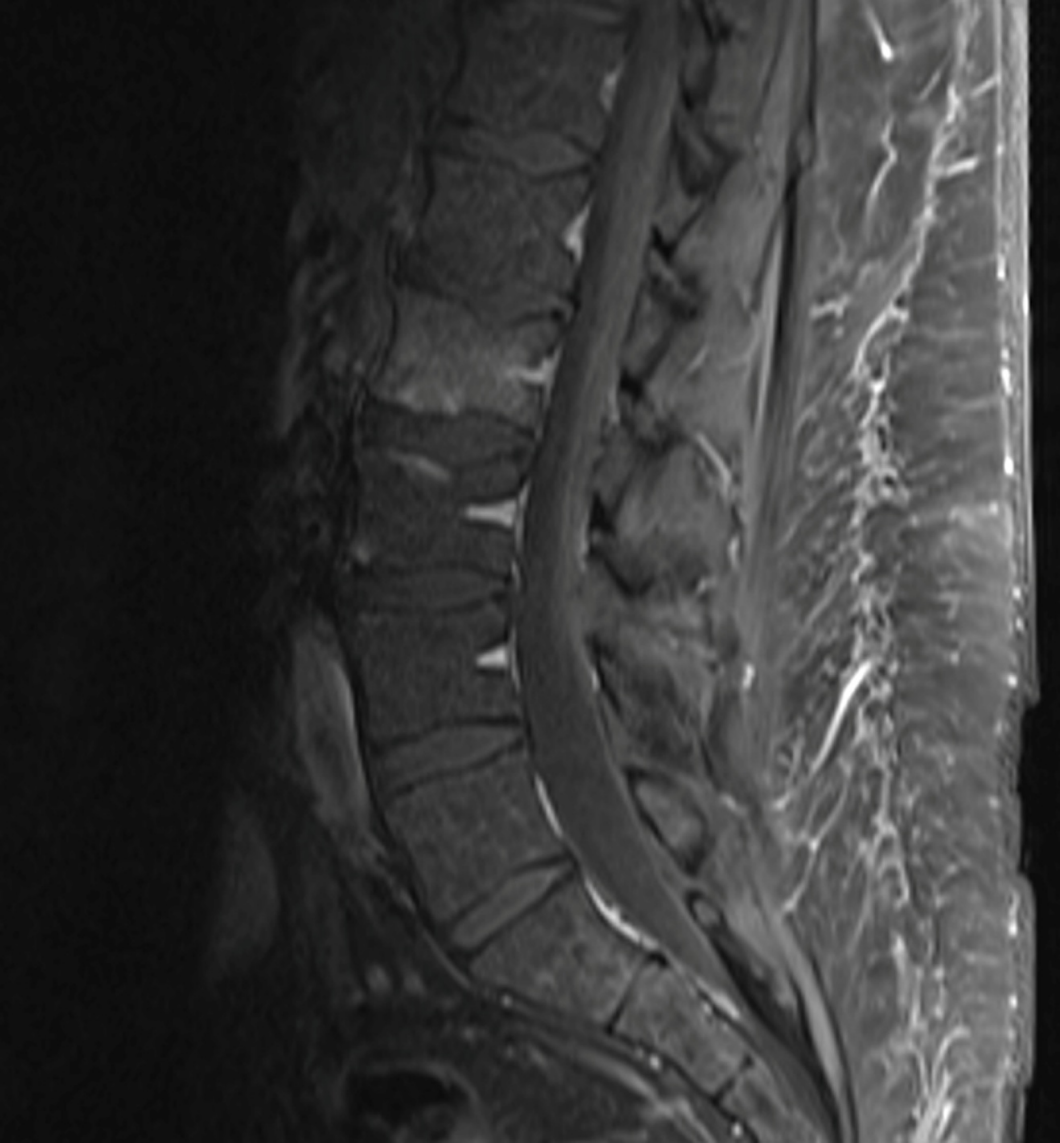
Initial MRI without contrast demonstrating foci of enhancement within the inferior L2 vertebral body.

Initial lumbar spine computed tomography (CT) obtained in the Emergency Department revealed a subtle irregularity of the L2 inferior endplate. Magnetic resonance imaging (MRI) of the lumbar spine again showed erosion of the L2 inferior endplate as well as diffuse L2 bone marrow edema, suggestive of either recent trauma or infection. MRI with contrast revealed small foci of enhancement of the L2 inferior endplate, as seen in Figures 1-3.

His laboratory studies revealed a white blood cell (WBC) of 18,000, erythrocyte sedimentation rate (ESR) 18, and a C-reactive protein (CRP) of 3.39. Peripheral blood cultures were negative. The patient was discharged with a thoracolumbosacral orthosis (TLSO) brace and instructions to return for repeat lab work and follow-up in clinic. 

The patient returned to the clinic one month later. He complained of continued, severe low back pain that significantly diminished his ability to ambulate. He also endorsed new bilateral lower extremity radiculopathy. The patient had attempted conservative measures with bed rest, the TLSO brace, and oral narcotics but had achieved only minimal pain relief.

**Figure 4:**
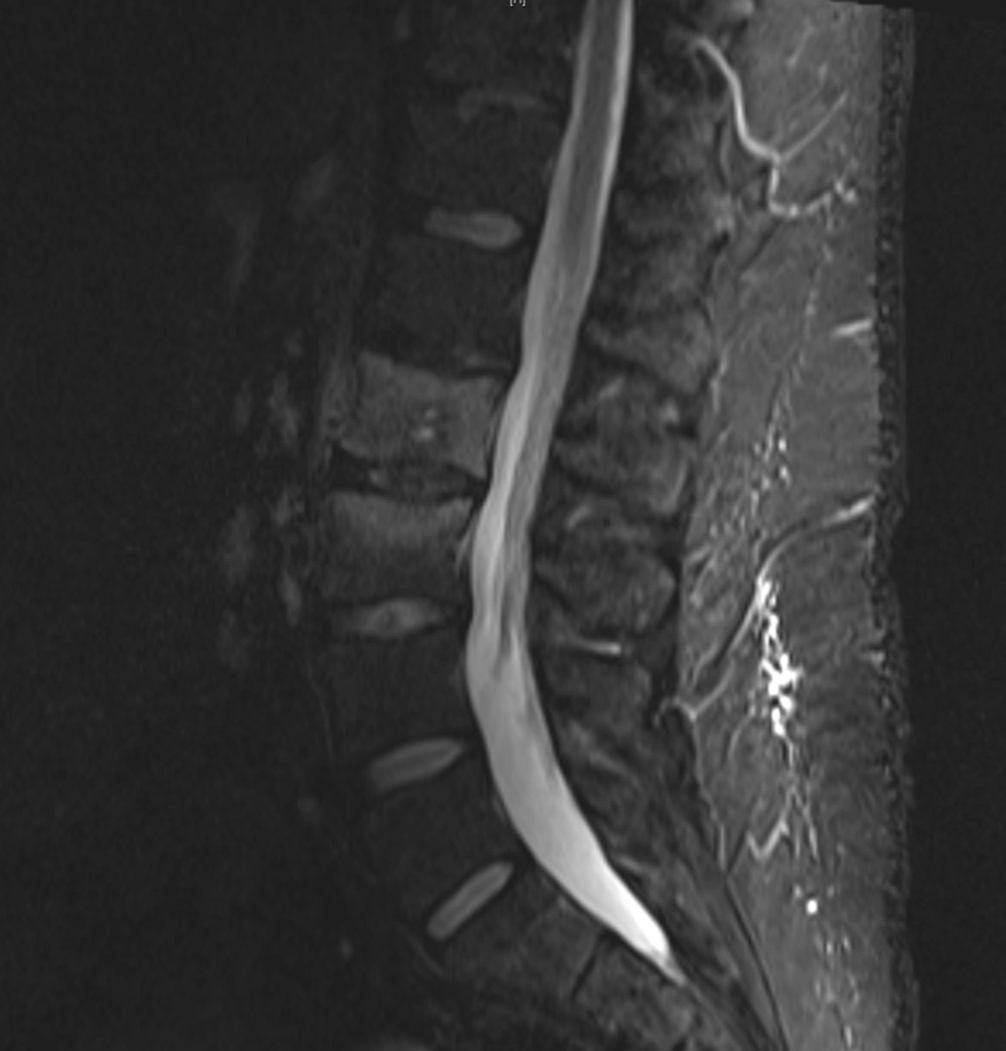
Follow-up MRI demonstrating increased signal change within the L2 vertebral body.

**Figure 5:**
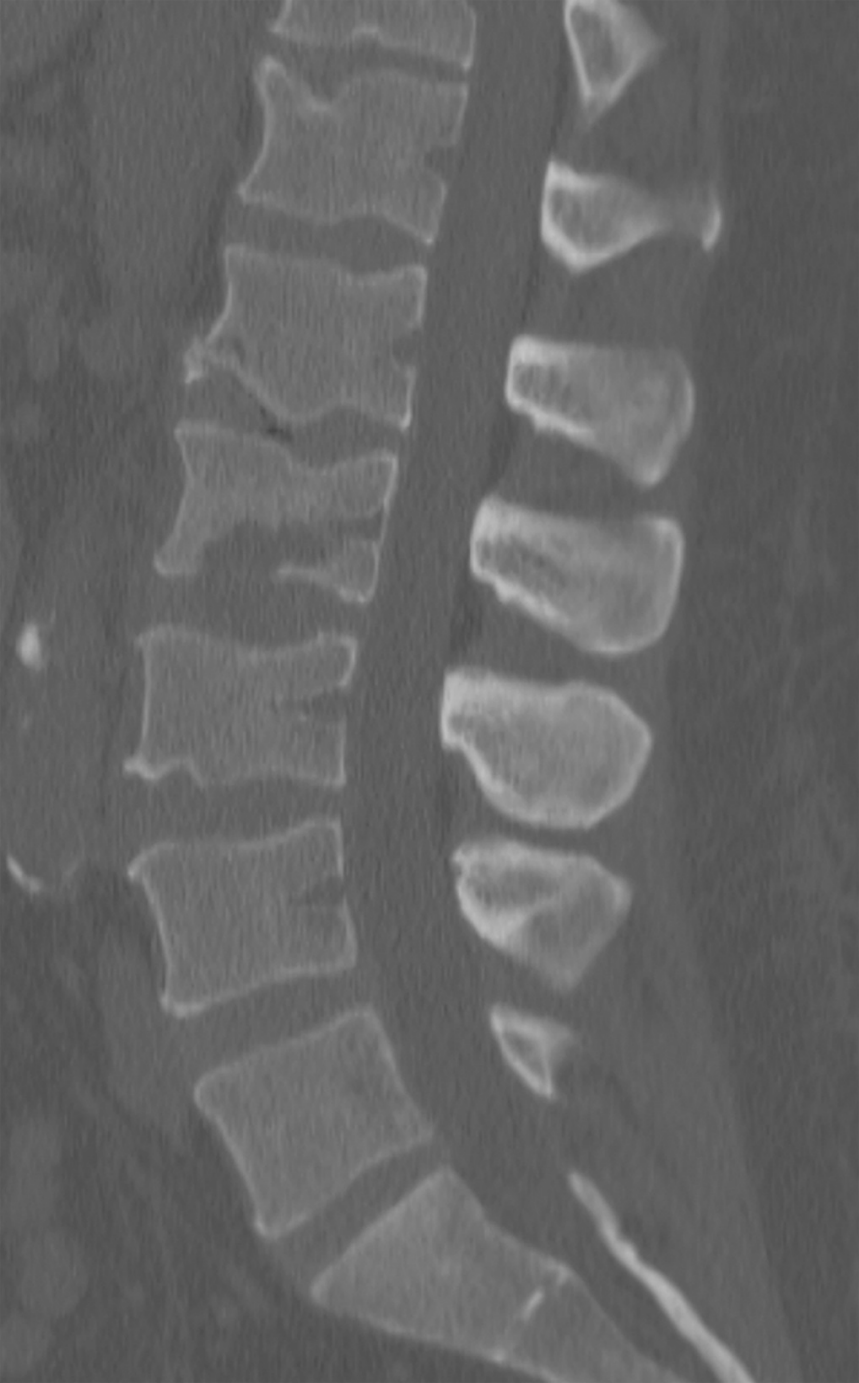
Follow-up CT demonstrating a significant progression of osseous destruction of the L2 vertebral body.

Repeat laboratory studies revealed an increased leukocytosis with WBC 20,600, an increased ESR to 29, and decreased CRP to 1.17. Repeat MRI and CT revealed a progression of the L2 central lytic lesion, as seen in Figures 4-5.

**Figure 6:**
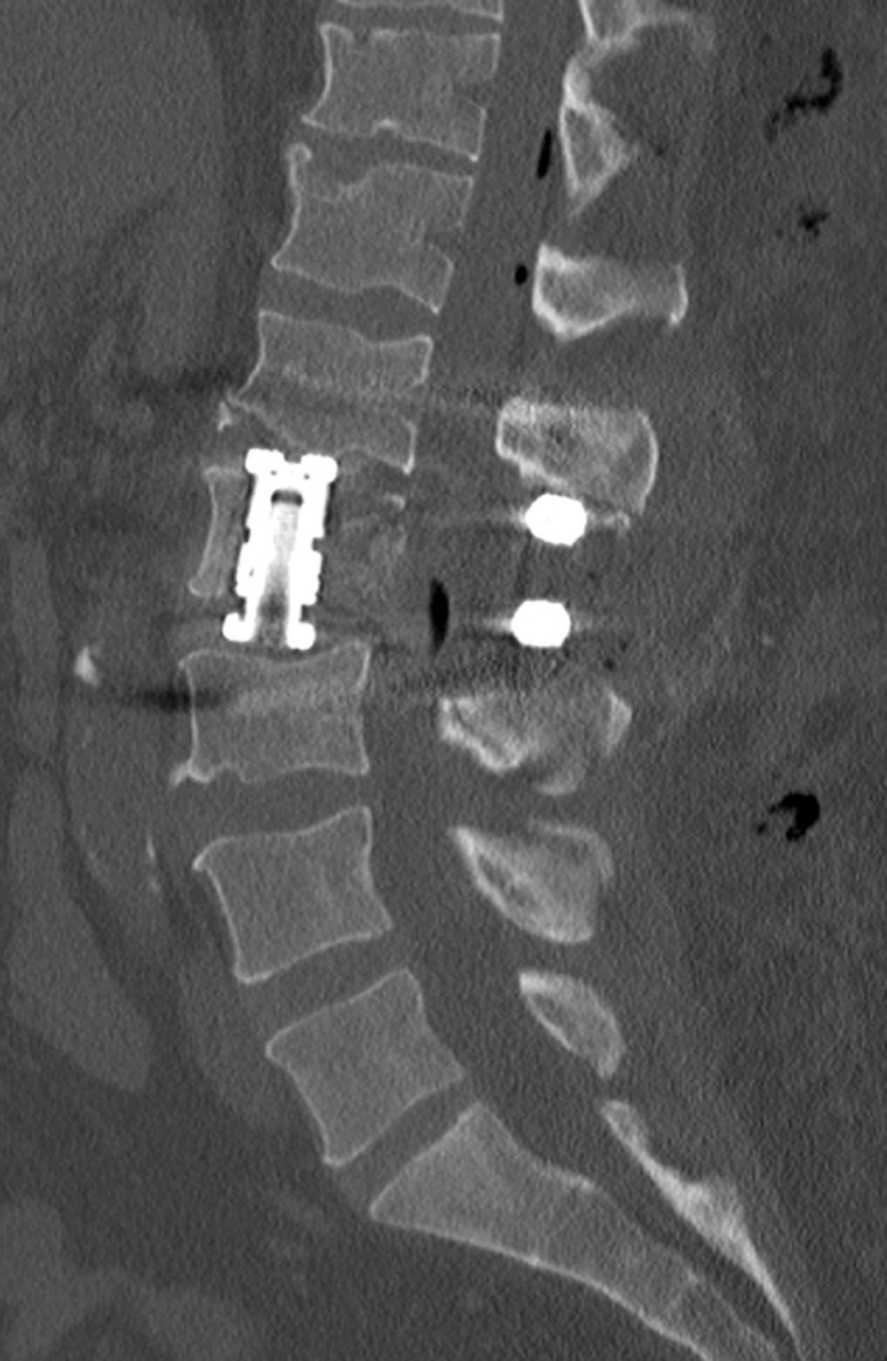
Postoperative CT shows interval corpectomy and placement of expandable cage.

The patient was admitted to hospital for definitive management. He underwent a CT-guided biopsy of the L2 vertebral body, which revealed *Granulicatella adiacens* on polymerase chain reaction (PCR). He subsequently underwent posterior decompression and fixation from L1 to L3 followed by L2 direct lateral corpectomy with the placement of an interbody cage. His postoperative film revealed appropriate resection of lytic L2 segments with complete neural element decompression (Figure 6). His intraoperative specimens did not reveal a positive pathogen on PCR but did produce NVS on culture, consistent with *Granulicatella adiacens*.

Preoperatively, the patient was started on vancomycin and ceftazidime after his biopsy. His antibiotics were narrowed to vancomycin after his surgical procedure. He was discharged to a skilled nursing facility on postop day six with plans for six weeks of IV antibiotics per the infectious disease team.

The authors report no conflict of interest concerning the materials or methods used in this study or the findings specified in this paper.

## Discussion

Predisposing factors to vertebral osteomyelitis include diabetes mellitus, intravenous drug abuse, advancing age, malnutrition, an immunocompromised state, malignancy, and chronic steroid usage [2]. Unfortunately, there is often a delay in diagnosis of several weeks to months [3]. Patients may not present with fever or elevated WBC; however, elevated inflammatory parameters, such a high ESR and CRP, are much more consistent [3]. Conservative measures, including antibiotic therapy, bracing, and, in certain cases, bed rest, are sufficient for treatment in the majority of cases [3-4]. In one study of 360 patients who met inclusion criteria for VO, 86% were treated successfully with conservative measures [4]. Surgical treatment is required for those with neurologic deficit, spinal instability, vertebral body collapse, progressive spinal deformity, or infection unresponsive to antibiotic therapy and conservative measures [3-6]. Complication rates from those patients requiring surgical intervention may be as high as 60% with up to a 25% reoperation rate [4]. Long-term functional studies show that most surgical patients fail to reach normal SF-36 physical function scores and are often associated with an adverse outcome [6].

The most common causative pathogens for vertebral osteomyelitis include *Staphylococcus aureus *and *Escherichia coli* [1, 3]. Here, we present a case of vertebral osteomyelitis caused by *Granulicatella adiacens*. Only four previous cases of VO caused by *G.* *adiacens* have been reported in the literature [7-09]. In 2000, NVS were subtyped into four categories, including *G. adiacens* [10]. NVS are normal flora of the upper respiratory, urogenital, and gastrointestinal tracts of humans. NVS do not grow in common culture media, and the sensitivity of isolation by conventional methods is low. Thus, diagnosis of these infections is often difficult. Furthermore, delayed treatment can lead to significant morbidity, as endocarditis is a common sequela [8].

Our patient presented with elevated WBC and ESR, but no fever. Given his recent history of trauma, his imaging was interpreted as possibly due to a mild compression fracture. His initial blood cultures were negative, and he was discharged without antibiotic therapy. The patient experienced significant progression of his VO in a four-week period, necessitating surgical intervention for stabilization.

## Conclusions

Vertebral osteomyelitis is a common entity that is most commonly managed successfully with conservative measures, including antibiotics and immobilization with bracing. Delays in diagnosis may lead to progressive osseous destruction necessitating surgical intervention, which is associated with a reasonably high complication rate. Medical practitioners should maintain a heightened awareness for uncommon pathogens and presentations of VO to reduce such delays. *Granulicatella adiacens* is one such rare cause of VO that is often difficult to culture on traditional mediums and often requires PCR methodology for isolation. Early isolation and treatment of pathogens, such as *G. adiacens*, may decrease the need for surgical intervention.
